# Impact of vitamin D supplementation on the clinical outcomes of COVID-19 pneumonia patients: a single-center randomized controlled trial

**DOI:** 10.1186/s12906-024-04393-6

**Published:** 2024-02-21

**Authors:** Pitchaya Dilokpattanamongkol, Chadakan Yan, Kulapong Jayanama, Pintip Ngamjanyaporn, Somnuek Sungkanuparph, Porpon Rotjanapan

**Affiliations:** 1 Department of Pharmacy, Faculty of Pharmacy, Mahidol University, Bangkok, Thailand; 2 Center for Clinical Epidemiology and Clinical Statistics, Faculty of Medicine, Chiang Mai University, Chiang Mai, Thailand; 3 Chakri Naruebodindra Medical Institute, Faculty of Medicine Ramathibodi Hospital, Mahidol University, Samut Prakan, Thailand; 4 Division of Allergy, Immunology and Rheumatology, Department of Medicine, Faculty of Medicine Ramathibodi Hospital, Mahidol University, Bangkok, Thailand; 5 Division of Infectious Diseases, Department of Medicine, Faculty of Medicine Ramathibodi Hospital, Mahidol University, Bangkok, Thailand

**Keywords:** Vitamin D, SARS-CoV-2, COVID-19, Pneumonia, Alfacalcidol

## Abstract

**Background:**

Vitamin D supplementation for infectious diseases has been discussed, but its role in COVID-19 is unclear. Therefore, this study examined the clinical outcomes of COVID-19 pneumonia patients who received vitamin D supplementation.

**Methods:**

This prospective, open-label, randomized controlled trial was conducted in a university hospital between July 2020 and March 2022. The inclusion criteria were patients aged ≥ 18 years with COVID-19 pneumonia patients. The patients were randomized into two groups: an intervention group receiving vitamin D supplementation (alfacalcidol, two mcg orally daily) until discharge and a control group. The clinical outcomes were pneumonia treatment duration, length of hospital stay, and change in pneumonia severity index between enrollment and discharge. Subgroup analysis was conducted for supplemental oxygen use, high-dose corticosteroid administration, evidence of lymphopenia, C-reactive protein concentration, and total serum vitamin D concentration. Adverse events were monitored.

**Results:**

Two hundred ninety-four patients were recruited (147 per group). The two groups did not differ in pneumonia treatment duration to discharge (*p* = 0.788) or length of hospital stay (*p* = 0.614). The reduction in the pneumonia severity index between enrollment and discharge was more significant in the intervention group (*p* = 0.007); a significant decrease was also observed among patients who had C-reactive protein > 30 mg/L (*p* < 0.001). No adverse reactions were recorded.

**Conclusions:**

Adding active vitamin D to standard treatment may benefit COVID-19 pneumonia patients who require supplemental oxygen or high-dose corticosteroid therapy or who have high C-reactive protein concentrations (> 30 mg/L) upon treatment initiation.

**Trial registration:**

Thai Clinical Trials Registry TCTR20210906005 (retrospectively registered, 6 September 2021).

**Supplementary Information:**

The online version contains supplementary material available at 10.1186/s12906-024-04393-6.

## Background

Coronavirus disease 2019 (COVID-19), caused by severe acute respiratory syndrome coronavirus 2 (SARS-CoV-2), was first identified in China at the end of December 2019. COVID-19 was declared a pandemic in 2020 and has caused more than 6.5 million deaths [1]. COVID-19 has led to an enormous public health crisis; it impacts physical and mental health and has increased morbidity and mortality rates worldwide [2]. Patients with COVID-19 can experience a wide range of clinical features, from asymptomatic disease to a systemic inflammatory response that induces lung injury [3], leading to poor clinical outcomes.

COVID-19 treatment includes supportive care and specific therapies, particularly in individuals at higher risk of severe disease. Supportive care comprises symptomatic treatment, intravenous fluid when dehydration is observed, and supplemental oxygen. Corticosteroids and other immunosuppressive agents, including baricitinib and tocilizumab, have been considered an essential therapy in more severe cases, particularly among individuals with evidence of desaturation in which a severe inflammatory process occurs. Specific therapies include molnupiravir, nirmatrelvir/ritonavir, remdesivir, neutralizing monoclonal antibodies, sotrovimab, and other newer antiviral agents. According to the WHO guidelines, favipiravir is an antiviral active against several types of RNA viruses, but it has not been approved for use as a primary COVID-19 monotherapy. However, due to the need for more specific treatment options in Thailand at the beginning of the pandemic, favipiravir was listed as an immediate COVID-19 treatment for local residents despite only limited evidence supporting its benefits [4]. The antiviral remdesivir was also available in Thailand during that time; however, a supply shortage limited its use to more severe cases. In Thailand, only favipiravir, remdesivir for severe cases, corticosteroids, immunosuppressive drugs, and other supportive measures were available for treating COVID-19 patients.

Immunization as a preventative modality mainly to alleviate the infection’s severity is another significant means to consider. Adequate vaccination against SARS-CoV-2 has been advocated worldwide to prevent severe disease and reduce mortality secondary to the infection. The Thai COVID-19 immunization scheme was launched in April 2020. Initially, the Sinovac-Coronavac COVID-19 vaccine was the only vaccine available to healthcare workers, while the ChAdOx1-S vaccine was available only to people older than 60 years. Immunization was available to the country's general population at the end of 2020. Thus, the lack of sufficient immunization against COVID-19 and the limited available treatment options might explain the higher mortality among Thais at the beginning of the pandemic.

Supportive care in Thailand was crucial because specific treatments had limited availability. Several researchers have investigated potential treatments that would help the body contain and eventually eradicate the infection; these include hydroxychloroquine, ivermectin, and vitamin D supplementation. Based on the most recent clinical evidence, hydroxychloroquine and ivermectin are not recommended as COVID-19 treatments [5, 6]. However, the addition of vitamin D to the COVID-19 treatment regimen has yet to be thoroughly analyzed regarding clinical benefits and adverse events. However, several clinical studies have shown a potential effect on critically ill individuals.

Low serum vitamin D concentrations generally impact musculoskeletal health and calcium homeostasis. However, vitamin D is also essential to the human immune system. 25OHD is an inactive form of vitamin D and does not impact the immune system. 25OHD-1α-hydroxylase in adipose tissue and various organs transforms 25OHD into an active form of vitamin D called 1,25-dihydroxy vitamin D (calcitriol). The kidney is the primary organ that produces calcitriol in humans [7]. Then 1, 25-dihydroxy vitamin D promotes antimicrobial activities of the innate cells, particularly macrophages and monocytes, as the next step [8–10]. Alfacalcidol can be considered an active vitamin D that acts as a prohormone of calcitriol. Alfacalcidol is cheap and has been discovered to have similar efficacy to calcitriol, especially in osteoporosis treatment, and is widely available in most hospitals in Thailand [11]. The highest dose of alfacalcidol that has been given in adults is two mcg per day (0.05 mcg/kg/day) continuously for two weeks and did not appear to cause any adverse events [12].

Besides its impact on musculoskeletal health, several clinical studies have demonstrated that vitamin D positively impacts the human immune system, especially in individuals with infectious diseases [13].

There is no consensus on whether vitamin D would improve COVID-19 treatment outcomes. Some clinical studies identified some clinical benefits of vitamin D administration, and some did not. Dakotas et al. performed a cohort study on 105 COVID-19 patients. They discovered that the patients who were vitamin D deficient tended to develop cytokine storms during their illness and required more care [14]. Another study from Germany by Radujkovic et al. conducted a retrospective cohort study on 185 COVID-19 patients. It demonstrated that patients with vitamin D deficiency tended to require mechanical ventilation, and the mortality was higher than those with normal vitamin D levels [15]. Ling et al. enrolled 444 patients in a retrospective observational study and identified decreased COVID-19-associated mortality in the patients who received cholecalciferol during their illness. However, several prospective cohort studies did not document the clinical benefits of vitamin D administration. Murai et al. enrolled 240 COVID-19 patients in a multicenter parallel, double-blind, randomized controlled trial to explore the benefits of a single high dose of cholecalciferol as a treatment regimen but failed to display positive impacts on hospital stay, mortality, ICU admission, and need for mechanical ventilation [16]. Two more extensive prospective studies by Orchard et al. and Jevalikar et al. also did not find a significant correlation between vitamin D deficiency and the severity of COVID-19 or mortality. Among the patients who were vitamin D deficient and received cholecalciferol treatment, there was no significant clinical outcome improvement [17, 18].

The proposed mechanisms of vitamin D on the immune system against viral infections are via vitamin D signaling innate immune response leading to increased T-cell cytokine release, particularly by pro-inflammatory Th1 cells [19]. Consequently, intracellular pattern recognition receptors are stimulated. Upon entering the viruses into the human body, the interferon regulatory family, alongside AP-1 and NF-КB, initiate transcriptional responses and generate antiviral effector cells [20].

Due to the diverse clinical outcomes from several studies regarding the impact of vitamin D on COVID-19, most clinicians have agreed that further studies on a larger scale are necessary. Several factors that were hypothesized to influence the clinical outcomes during vitamin D administration as COVID-19 treatment were 1.) the appropriate doses of vitamin D, 2.) the proper vitamin D formulary, and 3.) the patient’s characteristics that may obtain the maximal benefits from the supplementation [21].

Moreover, a causal relationship between vitamin D supplementation and COVID-19 pneumonia outcomes has yet to be established. There is little data on vitamin D supplementation in SARS-CoV-2-infected patients with pneumonia; effective treatment for this patient group is limited. In this study, we hypothesized that a high-dose active form of vitamin D, alfacalcidol supplementation in COVID-19 patients with pneumonia, would improve clinical outcomes.

The primary objective of this work was to determine the impact of high-dose-vitamin D supplementation on the treatment outcomes of COVID-19 pneumonia patients. The treatment outcomes investigated were pneumonia treatment duration, length of hospital stay, and change in the pneumonia severity index (PSI) between admission and discharge. The secondary objectives were to explore the epidemiology of SARS-CoV-2-infected individuals with low plasma vitamin D concentrations and the severity of COVID-19 associated with plasma vitamin D concentrations.

## Methods

### Study design

A prospective, open-label, randomized controlled trial was conducted at Chakri Naruebodindra Medical Institute, a university hospital in Thailand, between July 2020 and March 2022. The inclusion criteria were patients aged ≥ 18 years with COVID-19 pneumonia. Some patients were diagnosed with pneumonia on admission, while others developed pneumonia later in their clinical course. The exclusion criteria were patients diagnosed with gastrointestinal disorders with malabsorption syndrome, sarcoidosis, parathyroid gland disease, active tuberculosis infection, kidney stones, hypercalcemia, pregnancy or postpartum breastfeeding, those who had been on palliative treatment within one year of the study, and patients who had a contraindication for or hypersensitivity to vitamin D supplementation. Before the initiation of the study, the study protocol and informed consent were approved by the Faculty of Medicine Research Ethics Committee, Ramathibodi Hospital, Mahidol University (approval no. MURA2020/862) and was also retrospectively registered at the Thai Clinical Trials Registry (TCTR20210906005) on 6 September 2021. The primary outcomes of pneumonia treatment duration, length of hospital stay, and the change in PSI from baseline were compared between the two groups. The secondary outcomes were subgroup analyses according to the need for supplemental oxygen, 25-hydroxyvitamin D (25OHD) concentration (< 12 and < 20 ng/mL), prednisolone administration (≥ 1 mg/kg/day), lymphopenia (absolute lymphocyte count, ALC < 1000 cells/mm^3^), and C-reactive protein (CRP) concentration (< 30, ≥ 30, ≥ 40, and ≥ 50 mg/L).

### Data and sample collection

Our pilot study determined the sample size; the patients in the intervention and the control group had shown a reduction in the change in PSI scores of 4.00 and 7.50 (standard deviation of 8.43 and 20.45), respectively. To detect a mean difference in the change in PSI score using two independent means with a type I error (alpha) of 0.05, a type II error (beta) of 0.2, and 80% power with equal allocation to two arms, 314 patients would be required in each arm of the trial. Therefore, to allow for a 10% dropout rate, 346 people per arm and 692 patients were anticipated for the study.

On the day of enrollment, each patient was randomized into either the intervention or control group using blocked randomization with a selected block size of four. Block randomization was a computer-generated random number list prepared by an investigator without background clinical information. The intervention group received oral alfacalcidol (2 mcg daily or < 0.05 mcg/kg/day) until the end of hospitalization in addition to standard care for COVID-19. The control group was provided with routine care without vitamin D supplementation. The following patient information was collected: demographic data, coexisting medical conditions, serum concentrations of calcium, phosphate, total 25OHD, and CRP, PSI score at enrollment and discharge, use of supplemental oxygen, and the need for corticosteroids and other immunosuppressive therapies to assess the severity of the infection during the hospital stay. PSI calculation required patient demographics, including age and sex, co-morbidities, physical examination, and laboratory investigations [22]. Serum phosphate and calcium concentrations were determined on days 1, 7, and 14 of hospitalization. Additional blood tests were performed for clinical signs of hypercalcemia or hyperphosphatemia as potential adverse effects of vitamin D supplementation. The treating physicians discontinued alfacalcidol when there was a risk of hypercalcemia or hyperphosphatemia. However, the study was halted prematurely when the availability of remdesivir and molnupiravir as the primary treatment among symptomatic patients in Thailand after March 2022 was not an issue, in which the study team hypothesized that such treatment might predominate the effect of vitamin D significantly compared to the prior era that only favipiravir was widely prescribed in the country.

### Data analysis

Descriptive analysis was used for the baseline characteristics. Continuous variables are expressed as means (standard deviation) or medians (interquartile range; IQR), and categorical variables as counts (percentage). The outcome data were analyzed using the Mann–Whitney U test to compare distributions across the groups, and the non-parametric Wilcoxon signed-rank test was used to compare within-group distributions. The statistical analyses were performed using SPSS (IBM Corp. released 2019, IBM SPSS Statistics for Windows, version 28.0. Armonk, NY), with a *p*-value < 0.05 indicating statistical significance.

### Definitions

- A COVID-19 diagnosis was defined as an individual with acute respiratory symptoms who was positive for SARS-CoV-2 by real-time polymerase chain reaction (RT-PCR).
- COVID-19 pneumonia was defined as an individual with a new diagnosis of COVID-19 who had abnormal chest imaging (either plain chest radiograph or computerized tomography of the chest) consistent with COVID-19 involvement.
- PSI is a predicting rule to justify community-acquired pneumonia prognoses in terms of morbidity, mortality, and the need for hospitalization [23]. The scoring has been compared with other severity scores, namely, CURB-65, qSOFA, and MuLBSTA, for predicting all-cause in-hospital death for patients with COVID-19 pneumonia. And PSI was the most sensitive tool, with a specificity of 72.2% [22].
- Optimal vitamin D serum concentrations were defined as 30–60 ng/mL 25OHD [24].
- Vitamin D insufficiency was defined as a serum 25OHD concentration of 20–29.99 ng/mL [24].
- Vitamin D deficiency was defined as a serum 25OHD concentration of 12–19.99 ng/mL [24].
- Severe vitamin D deficiency was defined as serum 25OHD < 12 ng/mL [24].

## Results

**Fig. 1:**
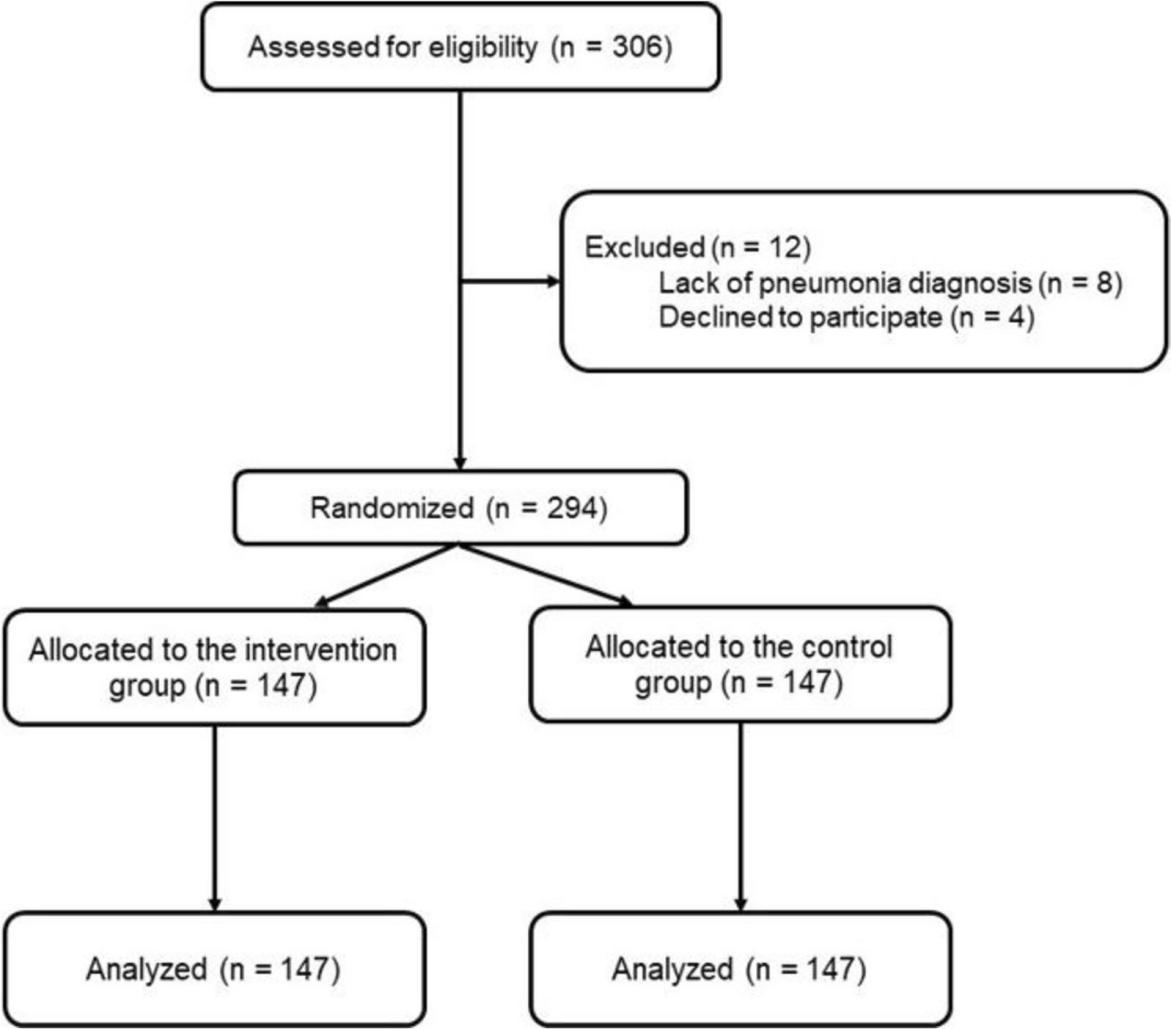
Patient flow diagram

In total, 306 COVID-19 patients were screened for enrollment; eight were excluded owing to a lack of pneumonia diagnosis, and four declined to participate in the study. A total of 294 patients with COVID-19 pneumonia were enrolled; 147 individuals were included in the intervention group, and 147 patients were included in the control group (Fig. 1). Table 1 presents the baseline characteristics of the enrolled patients.

**Table 1:** Baseline characteristics of enrolled patients

Characteristic	Intervention group (*n* = 147)	Control group (*n* = 147)	*p*-value
Sex, n (%)			0.129
• Men	62 (42.20)	75 (51.00)	
• Women	85 (57.80)	72 (49.00)	
Age (years), mean ± SD (min–max)	47.90 ± 16.77 (17–88)	53.71 ± 18.80 (19–91)	0.006
Weight, mean ± SD (min–max)	70.92 ± 16.83 (40.00–128.00)	71.05 ± 21.85 (38.50–165.00)	0.954
Height (cm), mean ± SD (min–max)	162.09 ± 8.54 (145.00–190.00)	161.78 ± 9.00 (140.00–185.00)	0.768
BMI (kg/m^2^), mean ± SD (min–max)	27.05 ± 5.62 (16.18–41.32)	26.81 ± 6.95 (0.00–50.93)	0.752
Serum calcium at enrollment (mg/dL), mean ± SD (min–max)	8.89 ± 0.48 (6.80–9.70)	8.98 ± 0.56 (6.80–10.50))	0.199
Serum phosphate at enrollment (mg/dL), mean ± SD (min–max)	3.54 ± 1.14 (2.20–13.00)	3.57 ± 0.73 (2.40–6.90)	0.819
Serum 25OHD at enrollment (ng/mL), mean ± SD (min–max)	22.50 ± 11.52 (7.31–56.27)	20.84 ± 13.59 (3.80–56.32)	0.269
PSI at enrollment, median (IQR)	46.00 (29.00)	50.00 (31.00)	0.045
PSI risk class, n (%)			0.549
• Low (≤ 90)	135 (91.84)	135 (91.84)	
• Moderate (91–130)	11 (7.48)	9 (6.12)	
• High (> 130)	1 (0.68)	3 (2.04)	
PSI risk class, n (%)			0.230
• < 50	86 (58.50)	75 (51.02)	
• 51–70	38 (25.85)	38 (25.85)	
• 71–90	11 (7.48)	22 (14.97)	
• 91–130	11 (7.48)	9 (6.12)	
• > 130	1 (0.68)	3 (2.04)	
Oxygen supplement, n (%)			0.756
• Ambient air	86 (58.50)	91 (61.90)	
• Cannula and noninvasive oxygen support	53 (36.05)	47 (31.97)	
• Endotracheal tube	8 (5.44)	9 (6.12)	
Steroid use, days			
• Number of patients	76	82	0.430
• Course steroid use, days, median (IQR)	7.00 (6.00)	6.00 (6.25)	
Medical conditions, n (%)			0.594
• Diabetes mellitus	24 (16.32)	23 (15.65)	
• Chronic kidney disease	2 (1.36)	4 (2.72)	
• Cerebrovascular disease	3 (2.04)	5 (3.40)	

*BMI* body mass index, *IQR* interquartile range, *PSI* pneumonia severity index, *SD* standard deviation, *25OHD* 25-hydroxyvitamin D

Approximately half of the patients in both groups were previously healthy; 24/147 patients (16.32%) in the intervention group and 23/147 (15.65%) in the control group had diabetes mellitus, all with reasonable diabetic control as indicated by glycated hemoglobin < 7.0%. The PSI was assessed in all patients, and most patients in both groups had PSI ≤ 90. None of the patients were vaccinated against COVID-19 prior to study enrollment.

At enrollment, 67/294 patients (22.79%) had lymphopenia (ALC < 1000 cells/mm^3^), of which 33/294 (11.22%) were in the intervention group and 34/294 (11.56%) in the control group. Additionally, 100/294 patients (34.01%) had CRP > 30 mg/L, of which 45/147 patients (30.61%) were in the intervention group, and 55/147 patients (37.41%) were in the control group. During hospitalization, 86/147 patients (58.50%) in the intervention group and 91/147 patients (61.90%) in the control group did not require supplemental oxygen. Fifty-three of 147 patients (36.05%) in the intervention group and 47/147 patients (31.97%) in the control group were on oxygen cannula or noninvasive ventilation. Furthermore, 8/147 patients (5.44%) in the intervention group and 6/147 patients (6.25%) in the control group had respiratory failure necessitating endotracheal intubation. Corticosteroids were prescribed in 76/147 patients (51.70%) in the control group and 82/147 patients (55.78%) in the intervention group. The median duration of corticosteroid use was seven days (IQR 6.00) in the intervention group and six days in the control group (IQR 6.25). All patients received favipiravir as an antiviral therapy except for 17 intubated patients who were given remdesivir as antiviral therapy.

The plasma vitamin D concentration was measured in 241 patients (Table 2). The mean serum 25OHD concentration at enrollment was 22.50 ± 11.52 ng/mL in the intervention group and 20.83 ± 13.58 ng/mL in the control group. Sixty-four of the 241 patients (26.56%) had vitamin D deficiency, and 13/241 (5.39%) had severe vitamin D deficiency. However, the majority of the patients having vitamin D insufficiency (102/105 patients (97.14%)), vitamin D deficiency (57/64 patients (89.06%)), or severe vitamin D deficiency (12/13 patients (92.31%)) had a low PSI at enrollment.

**Table 2:** Vitamin D deficiency and PSI at enrollment (241 patients)

Vitamin D deficiency	PSI at enrollment, n (%) (*n* = 241)		
	**Low, ≤ 90**	**Moderate, 91–130**	**High, > 130**
**Optimal vitamin D (30–60 ng/mL)**	50 (20.75)	8 (3.32)	1 (0.41)
**Vitamin D insufficiency (20–29.99 ng/mL)**	102 (42.32)	3 (1.25)	0 (0.00)
**Vitamin D deficiency (12–19.99 ng/mL)**	57 (23.65)	6 (2.50)	1 (0.41)
**Severe vitamin D deficiency (< 12 ng/mL)**	12 (4.98)	1 (0.41)	0 (0.00)

*PSI* pneumonia severity index

The pneumonia treatment durations are presented in Supplementary Fig. 1. The median pneumonia treatment duration to discharge was 7.00 days (IQR 5.00) in the intervention group and 7.00 days (IQR 4.00) in the control group (*p* = 0.788). The median length of hospital stay was 9.00 days (IQR 7.00) in the intervention group and 8.00 days (IQR 5.00) in the control group (*p* = 0.614). The two groups had no significant difference in these outcomes (Supplementary Fig. 2).

The PSI values of both groups at enrollment and discharge are shown in Supplementary Fig. 3. The reduction in PSI between enrollment and discharge was significantly greater in the intervention group than in the control group (*p* < 0.007). The median PSI of the intervention group was 46.00 (IQR 29.00) at enrollment and decreased significantly at discharge to a median PSI of 43.00 (IQR 27.00) (*p* < 0.001). In contrast, there was no significant difference between the PSI at enrollment (median 50.00, IQR 31.00) and discharge (median 48.00, IQR 34.00) in the control group (*p* = 0.679).

**Fig. 2:**
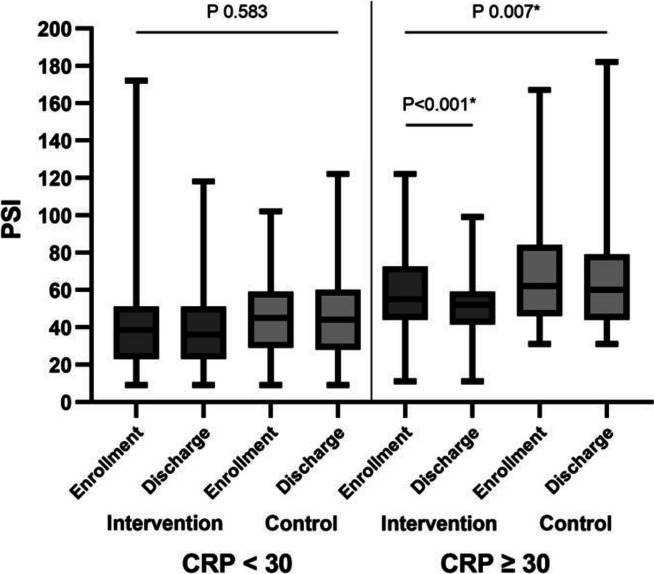
Pneumonia severity index (PSI) of intervention and control group patients with CRP < 30 mg/L and CRP over 30 mg/L

Table 3 presents the subgroup analyses of the clinical outcomes for the intervention and control groups. In patients who received supplemental oxygen, the median length of hospital stay was 12.00 days (IQR 8.50) in the intervention group and 11.00 days (IQR 9.50) in the control group. The median pneumonia treatment duration was 9.00 days in both groups (IQR 8.50 in the intervention group and 7.75 in the control group). The respective median PSIs at enrollment and discharge were 56.00 (IQR 32.50) and 52.00 (IQR 30.50) in the intervention group and 61.00 (IQR 39.00) and 60.00 (IQR 34.75) in the control group. The decrease in PSI between enrollment and discharge for patients who received supplemental oxygen was significantly greater in the intervention group than in the control group (*p* = 0.030). There was no significant difference between the intervention and control groups in hospital length of stay or in the reduction of PSI from enrollment to discharge for those with severe vitamin D deficiency. For those who were treated with corticosteroid therapy (prednisolone 1 mg/kg/day or higher), there was no significant difference in hospital length of stay between the two groups. However, among individuals who received prednisolone, those in the intervention group had a larger reduction in PSI, from 57.00 (IQR 29.00) at enrollment to 56.00 (IQR 28.00) at discharge (*p* = 0.008). Among the individuals with lymphopenia, adding vitamin D did not shorten the length of hospital stay or affect the PSI at discharge (Table 3). Among individuals with CRP ≥ 30 mg/L at enrollment, the addition of vitamin D reduced the median PSI from 55.00 at enrollment to 52.00 at discharge, which was a significant decrease (*p* < 0.001) (S1 Table). Among individuals with CRP ≥ 30 mg/L in the control group, the median PSI was reduced from 62.00 at enrollment to 60.00 at discharge (*p* = 0.459). The difference in PSI reduction between these two groups was statistically significant (*p* = 0.007), as shown in Fig. 2 and Supplemental Table 1. This phenomenon was also observed in individuals with higher initial CRP concentrations of 40 and 50 mg/L (*p* = 0.009 and *p* = 0.011, respectively) (Supplemental Table 1). There was no mortality among the study population.

**Table 3:** Clinical outcomes by subgroup analysis

**Any type of oxygen support**	**Intervention group** **(*n* = 61)**	**Control group** **(*n* = 56)**	***P*-value**
Pneumonia treatment duration, days, median (IQR)	9.00 (8.50)	9.00 (7.75)	0.835
Length of hospital stay, days, median (IQR)	12.00 (8.50)	11.00 (9.50)	0.893
PSI, median (IQR)			
PSI at enrollment	56.00 (32.50)	61.00 (39.00)	0.261
PSI at discharge	52.00 (30.50)	60.00 (34.75)	0.028*
* P*-value	< 0.001*	0.803	
PSI reduction from baseline	*P*-value 0.030*		
**Enrollment 25OHD < 12 ng/mL**	**Intervention group** **(*n* = 7)**	**Control group** **(*n* = 6)**	***P*-value**
Pneumonia treatment duration, days, median (IQR)	10.00 (17.00)	12.50 (14.75)	0.836
Length of hospital stay, days, median (IQR)	13.00 (17.00)	17.00 (18.00)	0.836
PSI, median (IQR)			
PSI at enrollment	57.00 (48.00)	69.00 (44.75)	0.534
PSI at discharge	43.00 (34.00)	68.50 (40.50)	0.101
* P*-value	0.059	0.317	
PSI change from baseline	**		
**Enrollment 25OHD < 20 ng/mL**	**Intervention group** **(*n* = 44)**	**Control group** **(*n* = 33)**	***P*-value**
Pneumonia treatment duration, days, median (IQR)	7.00 (9.50)	8.00 (4.50)	0.737
Length of hospital stay, days, median (IQR)	9.00 (10.50)	9.00 (5.00)	0.602
PSI, median (IQR)			
PSI at enrollment	40.50 (37.00)	56.00 (30.50)	0.024*
PSI at discharge	37.50 (32.50)	45.00 (35.50)	0.034*
* P*-value	0.023	0.056	
PSI change from baseline	*P*-value 0.799		
**Prednisolone dose ≥ 1 mg/kg/day**	**Intervention group** **(*n* = 39)**	**Control group** **(*n* = 46)**	***P*-value**
Pneumonia treatment duration, days, median (IQR)	9.00 (8.00)	9.00 (6.50)	0.114
Length of hospital stay, days, median (IQR)	13.00 (9.00)	10.50 (7.00)	0.068
PSI, median (IQR)			
PSI at enrollment	57.00 (29.00)	60.00 (32.50)	0.537
PSI at discharge	56.00 (28.00)	60.00 (33.25)	0.075
* P*-value	0.008*	0.882	
PSI change from baseline	*P*-value 0.060		
**Lymphopenia (ALC < 1000 cells/mm**^**3**^**)**	**Intervention group** **(*n* = 33)**	**Control group** **(*n* = 34)**	***P*-value**
Pneumonia treatment duration, days, median (IQR)	6.00 (5.00)	7.00 (4.25)	0.138
Length of hospital stay, days, median (IQR)	7.00 (8.50)	8.00 (5.00)	0.317
PSI, median (IQR)			
PSI at enrollment	58.00 (36.50)	57.50 (35.75)	0.494
PSI at discharge	56.00 (33.50)	51.00 (41.75)	0.940
* P*-value	0.050	0.784	
PSI change from baseline	*P*-value 0.120		

*ALC* absolute lymphocyte count, *IQR* interquartile range, *PSI* pneumonia severity index, *25OHD* 25-hydroxyvitamin D

^*^p-value < 0.05

^**^unable to determine *p*-value owing to small sample size

All patients in the intervention group tolerated vitamin D supplementation well, and no reports of diarrhea or feeding intolerance that might have affected vitamin D absorption were recorded. There were no adverse drug reactions documented during the study. The mean calcium concentration at enrollment was 8.89 ± 0.48 mg/dL in the intervention group and 8.98 ± 0.56 mg/dL in the control group. The mean serum phosphate concentrations were 3.54 ± 1.14 mg/dL and 3.57 ± 0.73 mg/dL in the intervention and control groups, respectively. There was no significant increase in serum calcium or phosphate concentrations in either group compared to the baseline calcium and phosphate concentrations.

## Discussion

SARS-CoV-2 has been a major public health threat worldwide for the past few years. Since 2019, there have been almost 600 million COVID-19 cases and more than 6 million deaths associated with SARS-CoV-2 infection [25]. As of August 2022, approximately 4.6 million COVID-19 patients and almost 32,000 deaths have been reported in Thailand, and these numbers continue to rise [25]. At the pandemic's beginning, COVID-19 patients primarily received symptomatic treatment because no specific antiviral agents nor SARS-CoV-2 vaccine was available. The mortality was higher among individuals with multiple comorbidities, e.g., obesity, older age, and pre-existing pulmonary conditions [26–28]. Per the national guidelines during the present study, all infected patients had to be hospitalized for treatment. A quarantine of at least ten days was required from the first day of symptom onset before the patient could be discharged.

The available specific treatment options in Thailand during the present study were antiviral therapies, namely, favipiravir for the majority of symptomatic patients and remdesivir for more severe patients, especially for those with respiratory failure requiring invasive ventilation. Alternative treatment modalities, or adjunctive care, have been discussed in Thailand, especially owing to concerns about inadequate vaccine distribution and a lack of specific antiviral medications. Hydroxychloroquine was initially of interest, but its use was later discouraged as more clinical data became available; the case was similar for ivermectin [29]. Vitamin D supplementation was considered owing to its safety profile and because it has been proven to impact the treatment of several infectious diseases positively.

Vitamin D is a hormone precursor that comes in two forms: 1) ergocalciferol or vitamin D2, and 2) cholecalciferol or vitamin D3. Humans acquire vitamin D from meat consumption and through synthesis processes when the skin is exposed to ultraviolet B. Vitamin D2 and D3 are delivered to the bloodstream and bind to the vitamin D binding protein. They are then transported to the liver, where vitamin D2, and D3 are processed into 25OHD, which is then deposited in the adipose tissue or transported to various organ systems. 25OHD is an inactive form of vitamin D and does not impact the immune system. 25OHD-1α-hydroxylase in adipose tissue and various organs transforms 25OHD into an active form of vitamin D called 1,25-dihydroxy vitamin D (calcitriol). The kidney is the primary organ that produces calcitriol in humans [7].

Vitamin D receptor (VDR) has been identified in multiple tissues (e.g., the peritoneum, uroepithelium, leukocytes, and lung tissue). VDR and calciferol interact to form a complex that triggers VDR gene transcription and produces cathelicidin and beta-defensin 4a, which have antimicrobial properties and promote the efficiency of white blood cells to eliminate fungal and viral pathogens [8–10]. Therefore, vitamin D has a role in the human immune system, particularly in infection prevention and the immune response.

In a recent survey among the Thai population, 45% were found to have either vitamin D deficiency or insufficiency (i.e., vitamin D serum concentration < 30 ng/mL), which might be even higher in urban settings, particularly in Bangkok, the capital city [30]. Moreover, patients with multiple comorbidities tended to have more severe vitamin D deficiency than the general population. Potential explanations for the observed deficiency are inadequate sunlight exposure, poor appetite, and hindered vitamin D absorption. Individuals with vitamin D deficiency reportedly have poorer clinical outcomes [31–33].

Overall, 294 COVID-19 patients were enrolled in our study. Previous studies have shown that COVID-19 patients with mild symptoms tend to have a good prognosis regardless of the treatment received [34–36], and this is consistent with our patients’ clinical manifestations. Most did not require supplemental oxygen during their clinical course and recovered entirely with supportive care and favipiravir, which is not listed as a primary antiviral agent for COVID-19 treatment. Older age, male sex, poorly controlled diabetes mellitus, coronary artery disease, chronic pulmonary disease, renal disease, and solid metastatic tumors are conditions with increased morbidity and mortality. However, only 6/294 patients (2.27%) included in our study had one of these conditions. This indicates that our study population did not include many patients who were at risk for severe COVID-19. There were statistically significant differences in age and PSI at enrollment. Importantly, no significant differences were observed when stratifying by PSI risk class. Furthermore, a comprehensive analysis of all parameters revealed no correlations between age and PSI at enrollment and outcomes. Based on the PSI, most patients were classified as having mild disease, and < 40% required supplemental oxygen and/or invasive ventilation. At enrollment, the baseline vitamin D concentrations were mainly in the insufficiency range rather than deficiency or severe deficiency. Only a few of the patients had severe vitamin D deficiency. Therefore, the study population’s characteristics were not different from the general urban Thai population, who may be slightly deficient in vitamin D. Thus, based on the current information, gradual recovery from SARS-CoV-2 infection without serious complications can be expected among the general population.

Among the patients with vitamin D deficiency or severe deficiency, supplementation with active vitamin D throughout their hospitalization did not impact the length of hospital stay or the duration of pneumonia treatment. This might have been because most patients had relatively mild diseases and did not require treatment or hospitalization longer than the duration stated in the national guidelines for COVID-19 treatment. However, a significant PSI reduction, mainly on the vital sign measurement (tachypnea, fever, and tachycardia) at discharge, was observed in intervention group patients with vitamin D deficiency and requiring supplemental oxygen. This phenomenon was not seen in patients with severe vitamin D deficiency, possibly because the study population in this category needed to be more significant. In patients with lymphopenia, adding active vitamin D to the treatment regimen did not shorten the duration of pneumonia treatment or length of hospital stay or reduce the PSI at discharge.

In individuals with more severe infections, especially those with pneumonia and respiratory distress, several reports have demonstrated an association between COVID-19 severity and elevated inflammatory markers, particularly CRP [37]. In this circumstance, corticosteroid therapy may be required, and a short treatment duration of < 10 days has been proposed to reverse the “excessive inflammatory response” so that the patient can restore appropriate oxygenation and eventually recover from respiratory distress [25, 38].

Our study observed a significant decline in PSI between enrollment and discharge in the intervention group in those with baseline CRP > 30 mg/L (*p* < 0.001). This decline in PSI was not observed in patients with CRP < 30 mg/L. This might be because COVID-19 impairs the function of type II pneumocytes, which decreases surfactant production; indeed, respiratory distress has been documented in many patients who died from COVID-19 pneumonia [39]. There is some evidence that vitamin D may enhance surfactant production [40]. This may explain why there was a significantly reduced PSI among the intervention group patients with more severe diseases. This finding was consistent with Alcala-Diaz et al., which conducted a retrospective, multicenter, non-randomized cohort study enrolling 537 patients and demonstrated that calcifediol administration after COVID-19 diagnosis reduced 30-day mortality [41]. Another retrospective study by Giannini et al. discovered that two doses of 200,000 IU of vitamin D on consecutive days could improve clinical outcomes regarding ICU admission and mortality in patients with multiple comorbidities [42]. Moreover, Sabico et al. recently performed a randomized controlled trial enrolling 69 patients with mild to moderate symptoms to receive 5,000 IU of vitamin D for two weeks. The intervention group recovered more rapidly from COVID-19. And at the end of the study, IL-6 levels decreased with treatment [43]. This may indicate that apart from corticosteroids, vitamin D doses qualify to lessen the inflammation in COVID-19 patients, in which excessive inflammation has been documented in more severe COVID-19 individuals. Therefore, a decline in PSI was observed only in patients with higher CRP levels.

In our study, 117 patients required supplemental oxygen, but corticosteroid therapy was prescribed to 158 patients. This may represent unnecessary corticosteroid use in 41/294 patients (13.95%). However, no harm that could be attributed to corticosteroid therapy was found in these individuals at discharge or when they returned to the hospital for follow-up six weeks later. In individuals with more severe hypoxemia, high doses of corticosteroids have been used intermittently with other immunosuppressive agents, e.g., tocilizumab and baricitinib [44]. In our study, 85/294 patients (28.91%) were prescribed a high dose of corticosteroids (> 1 mg/kg/day). A significant decline in the PSI at discharge was observed among these patients in the intervention group (*p* = 0.008), but no significant change was seen in the control group.

Among patients who received corticosteroid therapy, there were no significant differences between the intervention and control groups regarding the length of hospital stay, duration from pneumonia diagnosis to discharge, or duration from hospital admission to discharge. This could be explained by the national guidelines that recommended hospitalizing all COVID-19 patients for ten days from the first day of symptoms, regardless of the disease course. A significant decline in the PSI between enrollment and discharge was seen in the intervention group, whereas the PSI in the control group remained unchanged. This finding suggests that vitamin D can help to reduce the PSI when administered along with standard care, especially in patients who require supplemental oxygen, high-dose corticosteroid therapy, or who have serum CRP > 30 mg/L upon pneumonia diagnosis.

Our study had several limitations. First, a few of our participants had severe disease, and other risk factors associated with severe COVID-19 were not identified. Hence, spontaneous recovery was expected in these patients, and it might not have been possible to observe various aspects of improved clinical outcomes among the intervention group. Second, the nature of the COVID-19 pandemic situation sometimes prevented the treating physicians from obtaining laboratory tests per the study protocol timeline. However, this was only the case for some of the patients. Third, the national practice guidelines during the study period did not allow the treating physicians to discharge COVID-19 patients until day ten after symptom onset to help control the spread of SARS-CoV-2 infection. This circumstance limited an accurate assessment of the difference in hospitalization duration between the two groups; indeed, the study found no difference in the length of hospital stay between the groups. Fourth, the relatively small sample size may not allow us to determine the real benefits of vitamin D administration. The main reason for enrollment interruption was the change in antiviral prescription pattern from favipiravir-based to non-favipiravir-based (molnupiravir and remdesivir) that was more accessible, made an unfair comparison of the treatment outcomes between the initial phase of COVID-19 pandemic and the later stage in the country. Therefore, the study team decided to halt the study prematurely. Fifth, WHO classification for the disease severity stratification was not used in this study as the study ended before the existence of such classification.

However, this study highlighted the following important aspects:

1. Supplementing COVID-19 pneumonia treatment with high-dose vitamin D throughout hospitalization was safe and inexpensive (approximately 0.5 USD per day).
2. Unnecessary corticosteroid use was identified in this study.
3. Certain groups of COVID-19 pneumonia patients may benefit from high-dose vitamin D supplementation.

## Conclusions

Adding active vitamin D to the COVID-19 treatment regimen in our setting reduced the PSI at discharge when patients either required supplemental oxygen or high-dose corticosteroid therapy or had a high CRP concentration (> 30 mg/L) at treatment initiation. Vitamin D supplementation is considered a safe and low-cost treatment modality. It may be recommended for COVID-19 patients with pneumonia who have not responded well to routine immunization, particularly transplant patients, individuals who take immunosuppressive drugs, and older adults, to improve their clinical outcomes.

### Supplementary Information

**Additional file 1: Supplementary Figure 1. **Comparison of pneumonia treatment duration in the intervention and control groups.

**Additional file 2: Supplementary Figure 2. **Comparison of length of hospital stay in the intervention and control groups.

**Additional file 3: Supplementary Figure 3. **Comparison of pneumonia severity index (PSI) in the intervention and control groups.

**Additional file 4: Supplementary Table 1. **Clinical outcomes and PSI of intervention and control group patients with CRP <30, >30, >40, and >50 mg**/**L.

## Data Availability

The datasets generated during and/or analyzed during the current study are not publicly available but are available from the corresponding author on reasonable request and approval from the ethics committee.
