# Supplementary figures and images for: Impact of vitamin D supplementation on the clinical outcomes of COVID-19 pneumonia patients: a single-center randomized controlled trial
Source: BMC Complement Med Ther. 2024 Feb 21;24:97. doi: 10.1186/s12906-024-04393-6 (PMC10880207; doi:10.1186/s12906-024-04393-6)

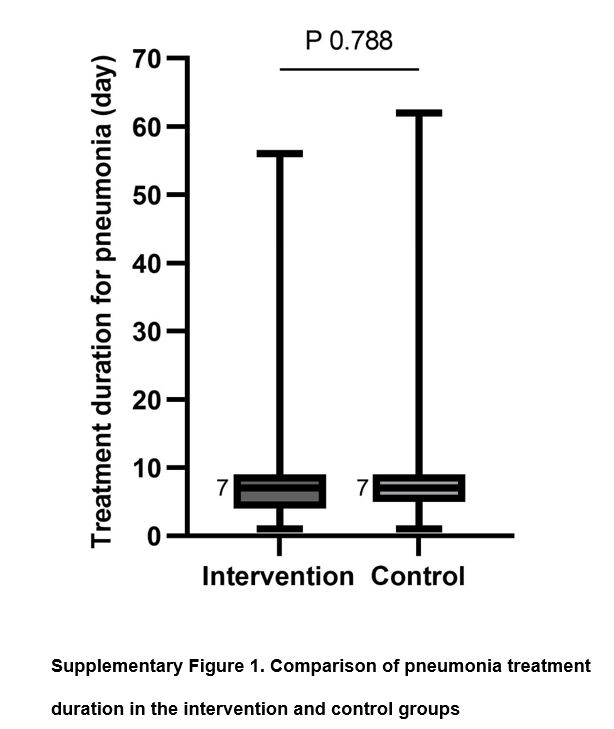

Supplement: Supplementary file 1 — Additional file 1: Supplementary Figure 1. Comparison of pneumonia treatment duration in the intervention and control groups. [file 12906_2024_4393_MOESM1_ESM.jpg]

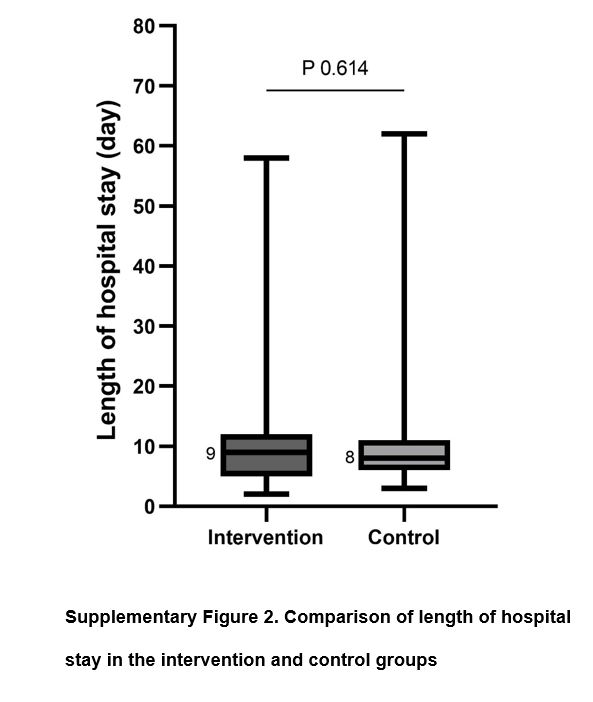

Supplement: Supplementary file 2 — Additional file 2: Supplementary Figure 2. Comparison of length of hospital stay in the intervention and control groups. [file 12906_2024_4393_MOESM2_ESM.jpg]

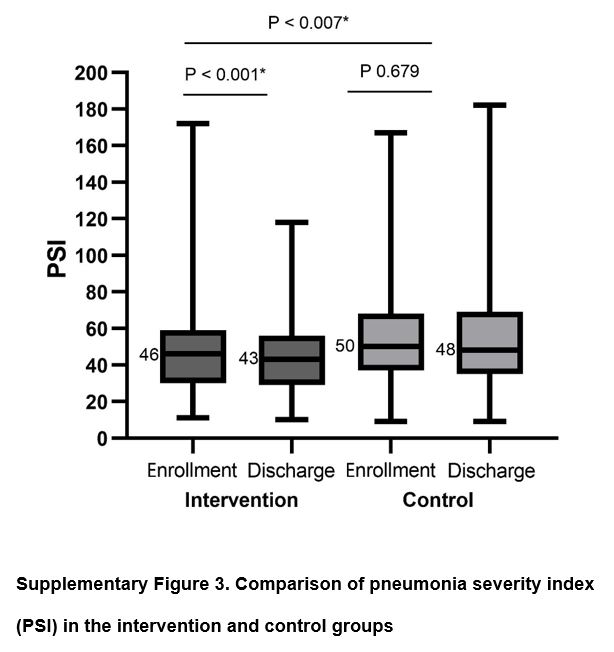

Supplement: Supplementary file 3 — Additional file 3: Supplementary Figure 3. Comparison of pneumonia severity index (PSI) in the intervention and control groups. [file 12906_2024_4393_MOESM3_ESM.jpg]
