# Supplementary material for: Impact of vitamin D supplementation on the clinical outcomes of COVID-19 pneumonia patients: a single-center randomized controlled trial
Source: BMC Complement Med Ther. 2024 Feb 21;24:97. doi: 10.1186/s12906-024-04393-6 (PMC10880207; doi:10.1186/s12906-024-04393-6)
Supplement: Supplementary file 4 — Additional file 4: Supplementary Table 1. Clinical outcomes and PSI of intervention and control group patients with CRP <30, >30, >40, and >50 mg/L. [file 12906_2024_4393_MOESM4_ESM.docx]

**Supplementary Table 1. Clinical outcomes and PSI of intervention and control group patients with CRP <30, >30, >40, and >50 mg/L.**

| **CRP <30 mg/L** | Intervention group  (n = 99) | Control group  (n = 87) | P-value |
| --- | --- | --- | --- |
| Pneumonia treatment duration, days, median (IQR) | 6.00 (4.00) | 6.00 (4.00) | 0.819 |
| Length of hospital stay, days, median (IQR) | 7.00 (5.00) | 8.00 (4.00) | 0.278 |
| PSI, median (IQR) |  |  |  |
| PSI at enrollment | 41.00 (31.00) | 45.00 (30.00) | 0.283 |
| PSI at discharge | 39.00 (29.00) | 44.00 (32.00) | 0.149 |
| P-value | 0.061 | 0.717 |  |
| PSI change from baseline | P-value 0.583 | |  |
| **CRP >30 mg/L** | Intervention group  (n = 45) | Control group  (n = 55) | P-value |
| Pneumonia treatment duration, days, median (IQR) | 9.00 (6.50) | 9.00 (8.00) | 0.754 |
| Length of hospital stay, days, median (IQR) | 11.00 (8.00) | 10.00 (8.00) | 0.385 |
| PSI, median (IQR) |  |  |  |
| PSI at enrollment | 55.00 (28.50) | 62.00 (38.00) | 0.066 |
| PSI at discharge | 52.00 (17.50) | 60.00 (35.00) | 0.010* |
| P-value | <0.001* | 0.459 |  |
| PSI change from baseline | P-value 0.007* | |  |
| **CRP >40 mg/L** | Intervention group  (n = 36) | Control group  (n = 47) | P-value |
| Pneumonia treatment duration, days, median (IQR) | 9.00 (7.75) | 9.00 (10.00) | 0.472 |
| Length of hospital stay, days, median (IQR) | 12.50 (9.00) | 10.00 (11.00) | 0.212 |
| PSI, median (IQR) |  |  |  |
| PSI at enrollment | 57.00 (28.50) | 63.00 (41.00) | 0.174 |
| PSI at discharge | 53.00 (18.25) | 61.00 (37.00) | 0.013* |
| P-value | <0.001* | 0.823 |  |
| PSI change from baseline | P-value 0.009* | |  |
| **CRP >50 mg/L** | Intervention group  (n = 32) | Control group  (n = 44) | P-value |
| Pneumonia treatment duration, days, median (IQR) | 9.00 (7.50) | 9.00 (9.50) | 0.580 |
| Length of hospital stay, days, median (IQR) | 11.50 (9.00) | 10.00 (10.25) | 0.287 |
| PSI, median (IQR) |  |  |  |
| PSI at enrollment | 56.50 (30.25) | 66.00 (36.75) | 0.072 |
| PSI at discharge | 52.00 (18.25) | 65.00 (37.00) | 0.004* |
| P-value | <0.001 | 0.823 |  |
| PSI change from baseline | P-value 0.011* | |  |

CRP, C-reactive protein; IQR, interquartile range; PSI, pneumonia severity index
* p-value < 0.05
